# Laparoscopic Cholecystectomy in Situs Inversus Totalis With Combined Indocyanine Green Fluorescence and Conventional Cholangiography: A Case Report

**DOI:** 10.7759/cureus.86761

**Published:** 2025-06-25

**Authors:** Kazuki Takeda, Kazuhiro Otani, Eiji Miyatake, Ryo Kohata, Masao Tanaka

**Affiliations:** 1 Department of Surgery, Shimonoseki City Hospital, Shimonoseki, JPN

**Keywords:** biliary tract imaging, “hepatobiliary surgery”, indocyanine green fluorescence, intraoperative cholangiography, laparoscopic cholecystectomy, mirror-image anatomy, situs inversus totalis

## Abstract

Situs inversus totalis (SIT), a complete form of situs inversus of the viscera, is a rare congenital anomaly that presents technical challenges during laparoscopic cholecystectomy (LC) due to mirror-image anatomy. We report a case of a 75-year-old female with known SIT and symptomatic cholelithiasis. After initial conservative management and endoscopic retrograde cholangiopancreatography (ERCP) for biliary sludge, elective LC was performed. A modified mirrored American-style port configuration was used, including a caudally placed left mid-clavicular port for right-hand dissection. Biliary navigation was enhanced by combining indocyanine green fluorescence cholangiography (ICG-FC) with conventional intraoperative cholangiography (IOC). ICG-FC allowed real-time identification of the cystic and common bile ducts, while IOC clearly delineated the common hepatic duct and confirmed the absence of residual stones. The operation was completed uneventfully in 108 minutes with 5 mL blood loss, and the patient was discharged on postoperative day three without complications. This case demonstrates that LC in SIT can be performed safely with meticulous preoperative planning, adapted port placement, and complementary imaging strategies. The integration of ICG-FC and IOC contributed to surgical safety by improving biliary visualization and minimizing the risk of injury.

## Introduction

Situs inversus of the viscera refers to a spectrum of rare congenital anomalies in which internal organs are located on the opposite side of the body. This spectrum includes situs inversus partialis (also known as incompletus), where only some organs are transposed, often accompanied by congenital heart defects, and situs ambiguus, which involves abnormal and inconsistent organ positioning and is frequently associated with severe anomalies such as asplenia or polysplenia. The most common subtype is situs inversus totalis (SIT), defined by a complete mirror-image arrangement of both thoracic and abdominal organs, with an estimated prevalence of one in 10,000 to one in 20,000 live births [1].

Laparoscopic cholecystectomy (LC) is the gold standard for treating symptomatic gallbladder disease [2,3]. However, in patients with SIT, the reversed anatomical configuration presents significant technical challenges. These include impaired spatial orientation and altered instrument ergonomics, particularly for right-handed surgeons, which may increase the risk of iatrogenic injury, most notably bile duct injury [2-4].

To mitigate these risks, intraoperative imaging plays a critical role. Indocyanine green (ICG) fluorescence cholangiography (ICG-FC) provides real-time, radiation-free visualization of bile-filled structures, while conventional intraoperative cholangiography (IOC) offers detailed anatomical mapping through contrast injection [5-7]. Several case reports have described the use of either ICG-FC [8,9] or IOC [10] in LC for patients with SIT. However, each modality has limitations when used alone: ICG-FC may not fully delineate the entire biliary tree or detect small calculi [11,12], whereas IOC, though comprehensive, can be technically cumbersome in a mirror-image setting. A combined approach using both ICG-FC and IOC has proven useful in other complex hepatobiliary cases, such as remnant gallbladder surgery involving accessory ducts [13]. Nonetheless, to our knowledge, their concurrent use during LC in a patient with SIT has not been previously reported.

To our knowledge, this is the first reported case of LC in a patient with SIT using a dual-imaging strategy combining ICG-FC and IOC. This case underscores the complementary strengths of both modalities in ensuring biliary safety and offers a novel framework for managing anatomical complexity in SIT.

## Case presentation

A previously healthy 75-year-old female with a history of hypertension and known SIT, diagnosed at age 18, presented to the emergency department with sudden-onset epigastric pain that had begun approximately one hour earlier. On admission, her vital signs were stable. Physical examination revealed tenderness in the epigastric and left hypochondriac regions without signs of peritoneal irritation. Laboratory tests demonstrated hepatobiliary inflammation, including marked elevations in transaminases and cholestatic enzymes, along with a mildly elevated white blood cell count, indicating a systemic inflammatory response (Table 1).

**Table 1:** Laboratory data at presentation.

Parameter	Result	Reference value
C-reactive protein (mg/dL)	0.15	0-0.14
Bilirubin, total (mg/dL)	1.2	0.4-1.5
Bilirubin, conjugated (mg/dL)	0.6	0-0.3
Aspartate aminotransferase (U/L)	788	13-30
Alanine aminotransferase (U/L)	453	7-23
Lactate dehydrogenase (U/L)	536	124-222
Alkaline phosphatase (U/L)	106	38-113
γ-glutamyltranspeptidase (U/L)	369	9-32
Blood urea nitrogen (mg/dL)	19.7	8-20
Creatinine (mg/dL)	0.71	0.46-0.79
White blood cell count (×10³/μL)	8.65	3.3-8.6
Hemoglobin (g/dL)	11.7	11.6-14.8
Platelet (×10³/μL)	222	158-348

**Figure 1:**
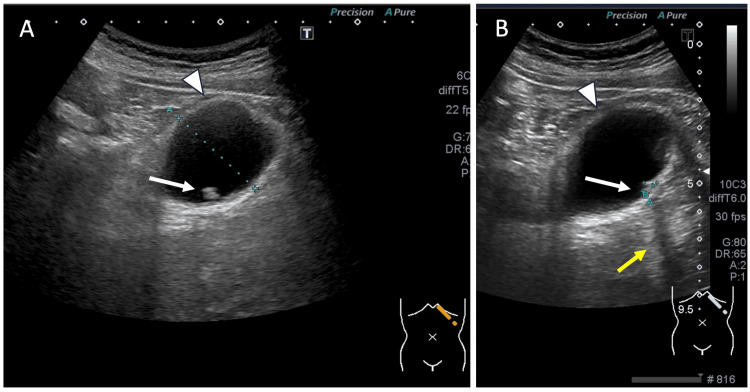
Abdominal ultrasound. A: An ultrasound image shows multiple hyperechoic lesions (white arrow) within the distended gallbladder (arrowhead). B: Another view from the same examination demonstrates posterior acoustic shadowing (yellow arrow), a characteristic feature of gallstones, although the calculus itself is partially obscured by the measurement cursor. Based on these findings, a clinical diagnosis of cholelithiasis was made (white arrow: multiple hyperechoic lesions, arrowhead: gallbladder).

**Figure 2:**
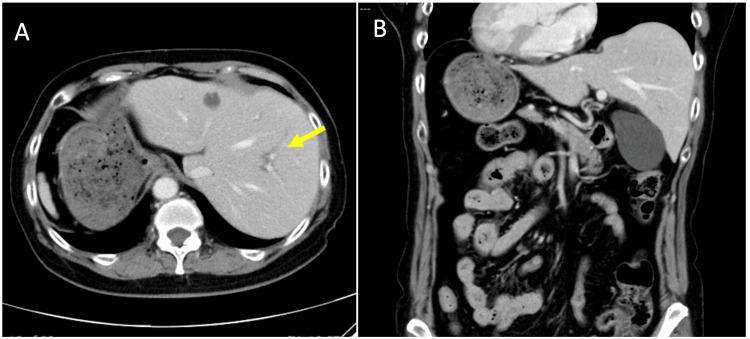
Contrast-enhanced abdominal computed tomography. A: Axial view showing a left-sided liver and right-sided stomach, consistent with mirror-image anatomy. Mild dilation of the intrahepatic bile ducts is also noted (yellow arrow). B: Coronal view confirming complete transposition of the thoracoabdominal organs, including dextrocardia.

**Figure 3:**
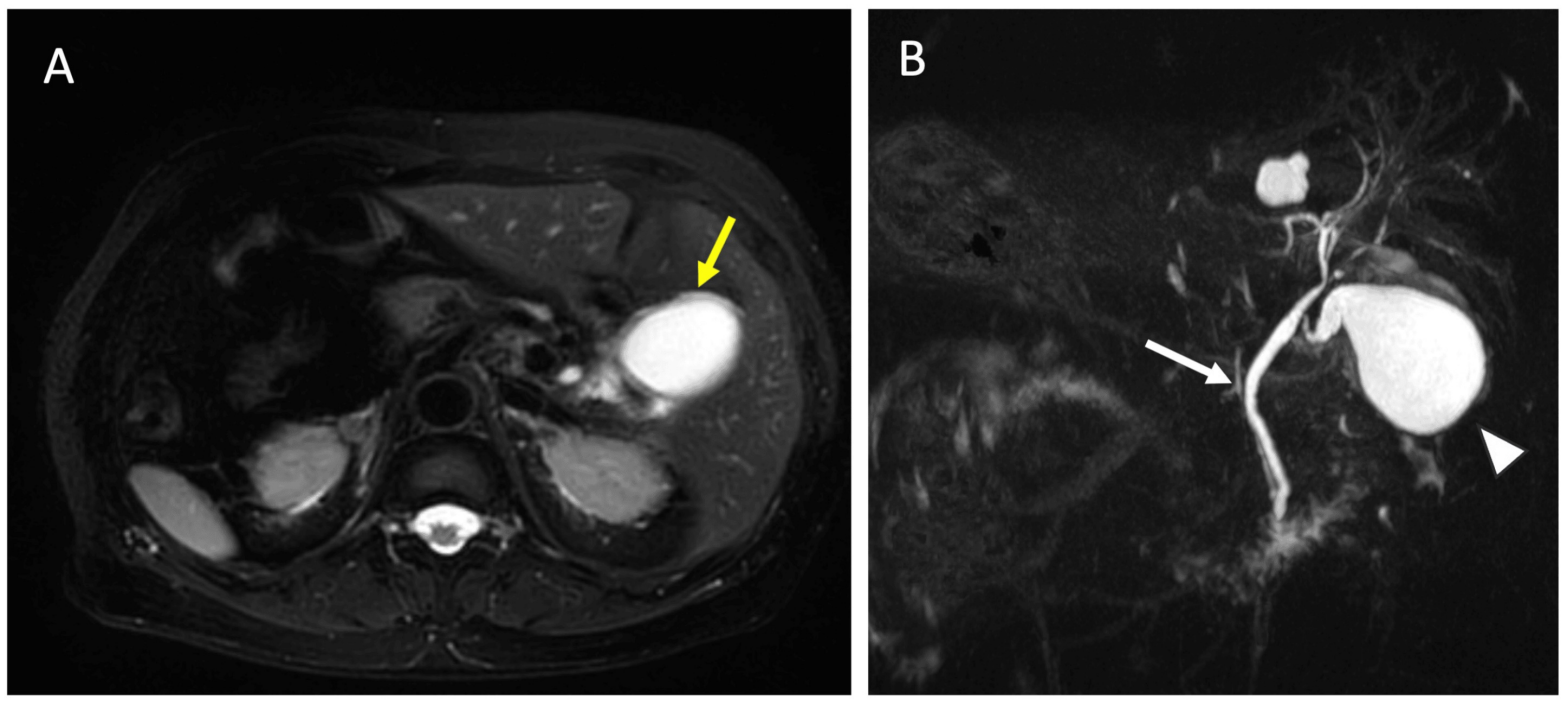
Magnetic resonance imaging. A: T2-weighted spectral presaturation with inversion recovery image shows mild pericholecystic edema (yellow arrow), consistent with acute cholecystitis. B: Magnetic resonance cholangiopancreatography (MRCP) delineates the biliary anatomy. Arrows indicate the gallbladder (arrowhead) and the common bile duct (CBD, white arrow). No definitive CBD stones or other structural abnormalities were identified.

Abdominal ultrasonography revealed multiple small gallstones (approximately 5 mm in diameter) within a distended gallbladder measuring 37 mm, along with a positive sonographic Murphy’s sign (Figure 1). Contrast-enhanced computed tomography (CT) confirmed the presence of SIT, revealing a complete mirror-image transposition of the thoracoabdominal viscera, including a left-sided liver and gallbladder, a right-sided stomach and spleen, and dextrocardia. The CT also showed mild dilation of the intrahepatic bile ducts, raising suspicion for a distal biliary obstruction such as choledocholithiasis, despite the absence of directly visualized stones (Figure 2). Magnetic resonance imaging (MRI), including MR cholangiopancreatography (MRCP), further confirmed SIT and revealed mild pericholecystic edema, consistent with acute cholecystitis, but no definitive common bile duct (CBD) stones or structural abnormalities of the biliary system were identified (Figure 3). 

**Figure 4:**
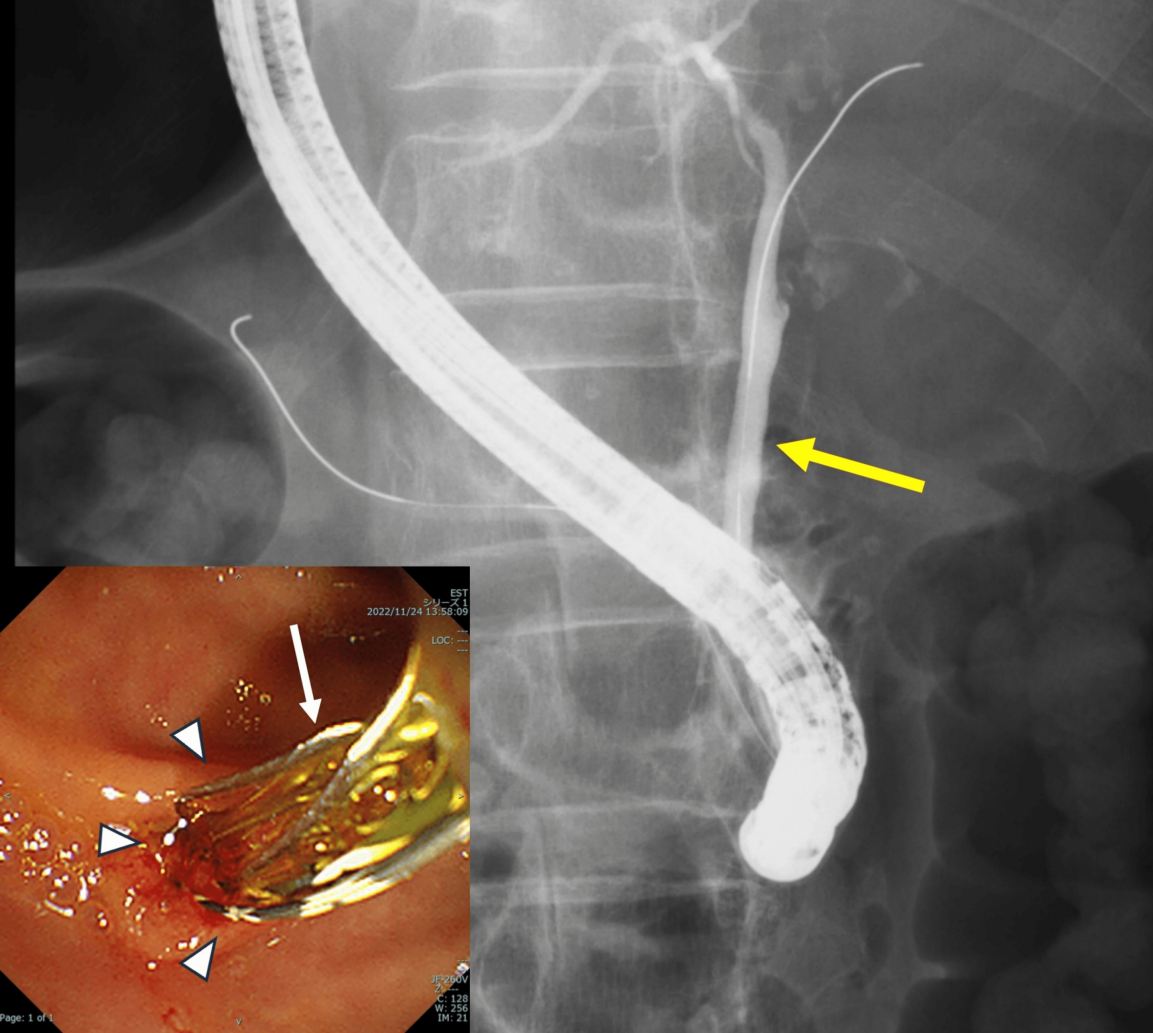
Endoscopic retrograde cholangiopancreatography. The main cholangiogram image shows the common bile duct (CBD), indicated by the yellow arrow. The inset provides an endoscopic view of the major duodenal papilla (arrowheads), where a basket catheter (white arrow) was used to successfully remove a small amount of biliary sludge.

Based on these findings, the patient was diagnosed with Grade I acute cholecystitis according to the Tokyo Guidelines 2018 [14], with suspected choledocholithiasis in the context of SIT. Initial management consisted of conservative treatment with intravenous antibiotics. Although her epigastric pain resolved by hospital day one, hepatobiliary enzymes remained elevated. Given the possibility of residual CBD stones and the heightened risk of recurrent cholangitis in patients with SIT, diagnostic and therapeutic endoscopic retrograde cholangiopancreatography (ERCP) with endoscopic sphincterotomy (EST) was performed on hospital day seven. A small amount of biliary sludge was successfully extracted from the CBD using a basket catheter (Figure 4). Following the procedure, the patient’s hepatobiliary enzyme levels decreased to near-normal levels, and she was discharged on hospital day 14 with plans for elective cholecystectomy.

In anticipation of the anatomical and technical complexities associated with SIT, the surgical team conducted a dedicated preoperative conference to develop a comprehensive operative strategy. During this process, CT and MRI images were meticulously reviewed to confirm the mirror-image configuration of the vascular and biliary anatomy and to rule out any additional anatomical anomalies. Based on these findings, a detailed surgical plan was established, which included a team-wide briefing to ensure shared situational awareness of the reversed anatomy; implementation of a mirrored American-style setup with ergonomic modifications for the right-handed surgeon, including caudal placement of the main working port; and a planned dual-imaging approach incorporating both ICG-FC and conventional IOC to enhance anatomical clarity and minimize the risk of biliary injury.

Following the resolution of acute inflammation, the patient underwent elective LC approximately three months after her initial presentation. For intraoperative biliary visualization, 2.5 mg of ICG was administered intravenously 27 minutes prior to skin incision. Near-infrared (NIR) imaging was performed using the Olympus VISERA ELITE II system (Olympus Corporation, Tokyo, Japan), incorporating the HD 3CMOS camera head (CH-S200-XZ-EB) and CLV-S200-IR light source.

**Figure 5:**
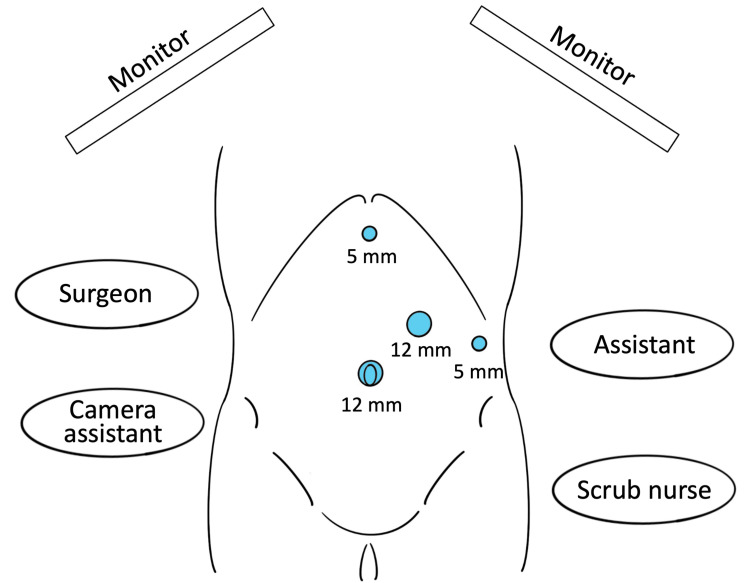
Adapted surgical positioning and trocar placement. Schematic illustration showing the mirrored American-style laparoscopic setup used for this case. The right-handed surgeon and camera assistant were positioned on the patient’s right side, with the assistant on the left. The 12-mm port placed at the left mid-clavicular line, three fingerbreadths below the costal margin, served as the main working port for the surgeon’s right hand, accommodating the reversed anatomy. Image created by the authors.

The surgical team employed a mirrored version of the standard American-style laparoscopic setup used at our institution: the right-handed surgeon and the camera assistant were positioned on the patient’s right side, with the assistant on the left. A 12-mm trocar was inserted at the umbilicus using the open (Hasson) technique. Three additional ports were placed as follows: a 5-mm epigastric port for the surgeon’s left hand; a 12-mm port located three fingerbreadths caudal to the left costal margin at the mid-clavicular line for the surgeon’s dominant right hand, strategically placed more caudally than usual to accommodate the reversed anatomy; and a 5-mm port at the left anterior axillary line for the assistant (Figure 5).

**Figure 6:**
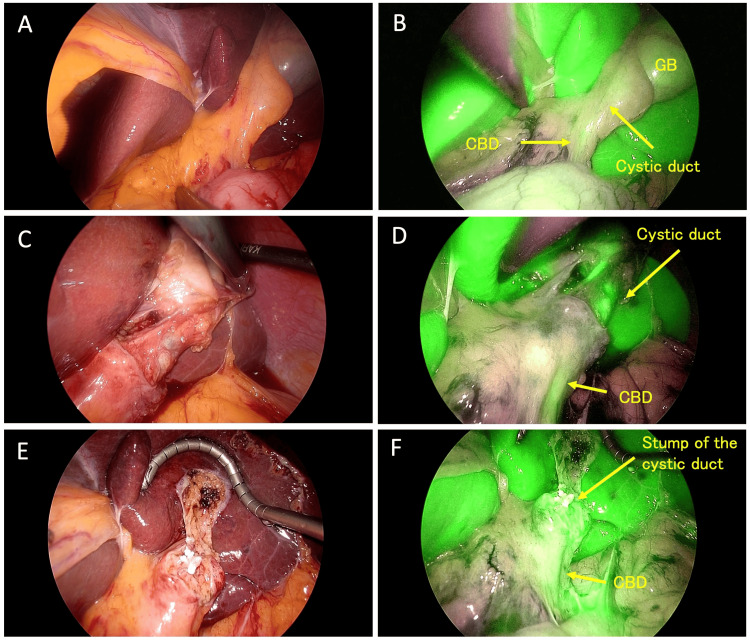
Laparoscopic views (white light and near-infrared fluorescence). Paired white light (A, C, E) and corresponding near-infrared fluorescence (B, D, F) images showing key operative stages. A, B: Initial view before dissection. Near-infrared imaging clearly identifies the gallbladder (GB), cystic duct, and common bile duct (CBD). C, D: View after dissection and achievement of the critical view of safety. The relationship between the cystic duct and the CBD is confirmed. E, F: Final view after gallbladder removal. The securely clipped stump of the cystic duct and the intact CBD are visualized, confirming the safety of the procedure.

Upon laparoscopic inspection, the gallbladder appeared grossly normal, without distention, wall thickening, or significant adhesions. ICG-FC enabled real-time identification of the cystic duct and its junction with the CBD through the hepatoduodenal ligament, aiding orientation in the mirror-image anatomy (Figure 6). However, the common hepatic duct was not clearly visualized with ICG-FC alone. The customized port configuration was generally effective for the right-handed surgeon, although the left-hand grasping instrument occasionally came close to the hepatoduodenal ligament, necessitating careful maneuvering.

**Figure 7:**
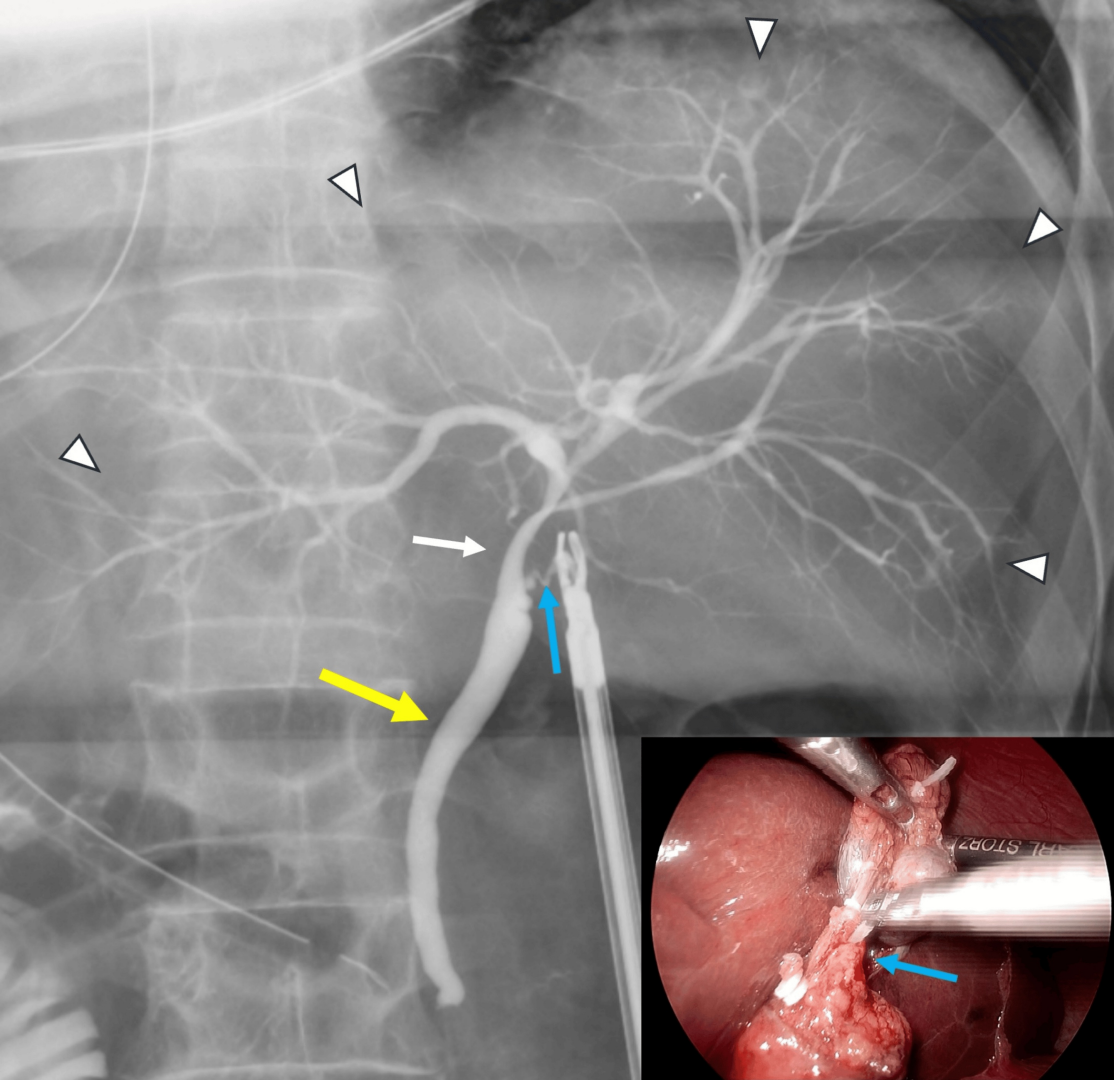
Intraoperative cholangiography (IOC). The main image shows the cholangiogram, confirming the biliary anatomy and ruling out retained stones. The key structures are identified as follows: intrahepatic ducts (arrowheads), the common hepatic duct (white arrow), the common bile duct (yellow arrow), and the cystic duct (blue arrow). The inset provides the laparoscopic view, showing the cannulation of the cystic duct to perform the cholangiography (blue arrow: cystic duct).

After achieving the critical view of safety, conventional IOC was performed using 30% meglumine diatrizoate (Urografin; Bayer Yakuhin, Ltd., Osaka, Japan) injected via a cystic duct catheter. IOC confirmed the absence of retained CBD stones and demonstrated intact biliary anatomy, including the common hepatic and intrahepatic ducts (Figure 7). The cystic artery and duct were clipped and divided, and the gallbladder was successfully removed.

The total operative time was 108 minutes, with an estimated blood loss of 5 mL. The postoperative course was uneventful, and the patient was discharged on postoperative day three without complications.

## Discussion

Gallstone disease is one of the most common digestive disorders worldwide, with a prevalence affecting approximately 10-15% of adults in developed countries and even higher rates reported in certain ethnic populations [15]. Although SIT is a rare congenital condition, there is no conclusive evidence that it increases susceptibility to gallstone disease. A recent review of 191 published cases of situs inversus reported a cholelithiasis prevalence of 13.6%, a figure comparable to that of the general population [16]. Nonetheless, cholelithiasis remains the most frequently encountered digestive pathology in SIT patients [16] requiring surgical attention. As such, understanding the diagnostic and operative considerations for gallstone disease in the context of SIT is of substantial clinical importance.

LC in patients with SIT presents distinctive anatomical and ergonomic challenges due to the mirror-image arrangement of abdominal organs, which disrupts spatial orientation and instrument handling, particularly for right-handed surgeons [2-4]. While some studies have suggested left-handed surgeons may hold an advantage regarding operative time [3,17], thoughtful port placement modifications are crucial for optimizing the performance of right-handed surgeons [18,19]. In a standard mirrored American-style setup for SIT, attempting right-handed dissection through the left mid-clavicular port while retracting with the left hand via the epigastric port often leads to problematic instrument interference. Our approach, similar to the principle described by Phothong et al. [10], involved a key modification: caudal repositioning of the left mid-clavicular dissecting port. This strategic adjustment significantly improved the ergonomics for the right-handed surgeon by creating a more favorable angle for dissection with the dominant hand, thereby reducing instrument interference and facilitating more intuitive movements relative to the left-sided gallbladder.

While this modification enhanced right-handed operability for the main dissection, the complexity of SIT meant that optimal anterior dissection occasionally still benefited from left-handed manipulation via the epigastric port by the primary surgeon, highlighting the value of ambidextrous capability. Furthermore, consistent and effective retraction by the assistant remained essential, a point emphasized by Du et al.'s recommendation for two assistants to further support the primary surgeon's dominant hand [20].

Our experience demonstrates that specific port modifications, such as the caudal shift of a key working port, offer substantial ergonomic benefits. These changes make the procedure more manageable. However, a universally standardized LC technique for SIT has yet to emerge. An individualized approach, leveraging such successful adaptations while maintaining intraoperative flexibility, remains critical.

Beyond ergonomic adaptations, achieving clear intraoperative visualization of the biliary anatomy is essential for minimizing the risk of complications during LC in patients with SIT. ICG-FC is increasingly recognized for its ability to provide real-time, non-invasive visualization of the biliary tree, offering a dynamic “road map” [6,21] that is especially valuable in anatomically complex scenarios such as SIT [8,9]. Its advantages include simplicity of use, absence of radiation exposure, and the ability to visualize biliary structures without requiring cannulation of the cystic duct [6,21]. In our case, 2.5 mg of ICG was administered intravenously 27 minutes prior to skin incision, consistent with commonly used short-interval protocols. Large-scale studies, including the Fluorescence-Assisted Laparoscopic Cholangiography (FALCON) randomized controlled trial [7], have adopted similar regimens (e.g., administration approximately 30 minutes preoperatively) and demonstrated benefits such as faster identification of biliary structures. However, the optimal timing of ICG administration remains under investigation. While short-interval protocols are practical and effective, some studies suggest that a longer interval, at least three hours, may optimize the bile duct-to-liver fluorescence ratio, thereby improving contrast and visibility [22]. At present, no standardized consensus exists, and the choice of timing may represent a trade-off between procedural convenience and image quality.

Despite its advantages, ICG-FC has well-documented limitations. The penetration depth of near-infrared light is limited to approximately 5-10 mm [23], which can hinder visualization in patients with obesity, dense inflammatory changes, or excessive perihepatic fat [11,21]. Furthermore, while ICG-FC highlights bile-containing structures, it is not suitable for detecting bile duct stones. In contrast, conventional IOC remains the gold standard for identifying choledocholithiasis and delineating the full biliary anatomy, including small or aberrant ducts [11,12]. 

Consistent with these limitations, the common hepatic duct was not clearly visualized by ICG-FC alone in our patient. Therefore, IOC was performed as a complementary imaging modality following initial dissection. It provided definitive confirmation of the complete biliary anatomy, including the common hepatic duct, and ruled out retained CBD stones, a crucial consideration given the preoperative suspicion and the prior ERCP for biliary sludge. Moreover, IOC offered final verification to prevent iatrogenic injury, which is of particular importance in the mirror-image anatomy of SIT. This dual-imaging strategy, using ICG-FC for initial real-time orientation and IOC for comprehensive anatomical confirmation, enhanced intraoperative safety. By minimizing the risk of biliary misidentification and injury, it proved to be a practical and robust approach for navigating the complex anatomy associated with SIT.

In our case, the total operative time was 108 minutes with minimal estimated blood loss (5 mL), and the patient had an uneventful postoperative course with no complications. Our operative time, while appearing longer than the mean of approximately 71-74 minutes reported in systematic reviews of LC in SIT [2-4], is comparable when procedural differences are considered. It is crucial to note that these published averages are heavily weighted by cases where routine IOC was not performed; for instance, a systematic review by Enciu et al. [3] found that IOC was conducted in only about 10% of reported cases, and another by Chaouch et al. [2] reported a rate of 17.2%. IOC is a time-consuming step, estimated to prolong surgery by 10 to 25 minutes. Therefore, our operative time, which deliberately included IOC as part of a dual-imaging strategy to ensure maximal safety, can be considered comparable to that of other reported cases. Our complication-free outcome aligns with the high safety profile reported for LC in SIT, which is characterized by very low morbidity (<1%) and near-zero conversion and mortality rates in experienced hands [2-4].

Our experience provides several important clinical insights. First, thorough preoperative imaging is essential not only to confirm SIT but also to detect any associated anatomical anomalies [1] that may alter the surgical plan. For example, Afifudin et al. [24] reported a case in which an undiagnosed SIT led to a Strasberg type E3 bile duct injury requiring open hepaticojejunostomy. Second, ergonomic adjustments, such as our caudally repositioned main working port, can mitigate technical difficulties and should be considered based on the surgeon’s handedness and institutional practices. Third, the complementary use of ICG-FC and IOC allows for enhanced biliary visualization and greater procedural confidence, particularly when anatomical landmarks are ambiguous or reversed. Sharing such technical refinements can contribute to the development of flexible, case-based strategies for managing rare anatomical configurations like SIT.

## Conclusions

LC for symptomatic cholelithiasis in patients with SIT, a rare congenital condition that poses significant anatomical and ergonomic challenges, is feasible and safe when supported by meticulous preoperative planning, customized ergonomic strategies, and the selective use of intraoperative imaging modalities. The dual application of ICG-FC and IOC in our case effectively compensates for individual limitations of each modality, enhancing surgical safety. This approach may serve as a practical model for the minimally invasive management of similarly complex anatomical variants.
